# Imaging the Entire Sexual Life Cycle of the Budding Yeast *Saccharomyces cerevisiae* Using a Microfluidic Platform

**DOI:** 10.21769/BioProtoc.5536

**Published:** 2025-12-05

**Authors:** Taylor Kennedy, Sandhya Neupane, Bryn Merritt, Orlando Argüello-Miranda

**Affiliations:** Department of Plant and Microbial Biology, North Carolina State University, Raleigh, NC, USA;

**Keywords:** Microbial life cycle, Microfluidics, Live-cell imaging, Sexual reproduction, Proliferation, Fungi, Yeast

## Abstract

Microbial life cycles are often reconstructed theoretically from fragmentary pieces of evidence. Protocols for the direct and continuous observation of entire microbial life cycles, including sexual reproduction, are scarce, which limits the study of cellular transitions between different life cycle stages and prevents the visualization of cryptic stages. Although sequence-based techniques, such as -omics approaches, can reconstruct cellular transitions at the genetic and biochemical level, these methods are destructive and do not recover information from the same living cell over time. This protocol provides a solution to directly and continuously observe microbial life cycles, including sexual reproduction, by using microfluidics manipulations that expose single cells to nutritional stimuli and selective pressures. As proof of principle, we triggered a life cycle sequence transition in the model yeast *Saccharomyces cerevisiae*, starting with an arrest of proliferation in an ancestor cell followed by induction of meiosis through starvation, selection of sexually reproducing cells through exposure to a drug cocktail, germination of haploid spores, and mating of haploid individuals, creating a new descendant generation. This protocol offers the possibility to directly compare molecular and cellular behavior across life cycle stages and across sexually reproducing generations.

Key features

• Outlines optimal microfluidic conditions for all life cycle stages in the model microorganism *S. cerevisiae*.

• Describes a selection method for sexually reproducing sporulated yeast cells and against quiescent cells.

• Provides descriptions for optimal loading of yeast cells in microfluidic devices for long-term imaging.

• Enables continuous detection across life cycle transitions, which could be applied to other biomedically and agriculturally relevant fungal microorganisms.

## Graphical overview


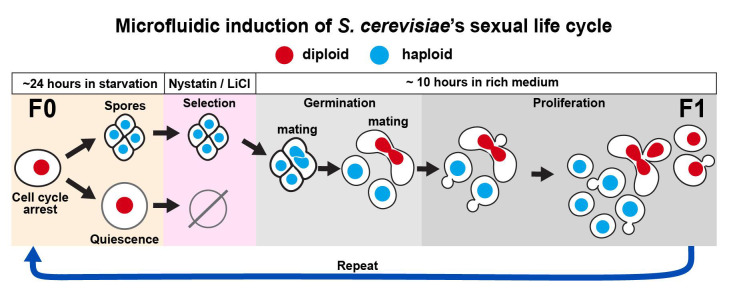


## Background

The life cycle of *Saccharomyces cerevisiae* has been used as a model system for studying all major transitions in eukaryotic organisms, ranging from cell division to sexual reproduction [1–3]. Historically, each life cycle stage has mostly been studied separately, and direct comparisons between life cycle stages are usually not possible because of different experimental requirements. For instance, protocols for studying meiosis require the use of synchronization methods, which affect cell cycle pathways, preventing a direct comparison of different cell states with minimal perturbations. As a result, descriptions of the yeast life cycle largely rely on fragmentary genetic and microscopy evidence instead of direct and continuous observations, a problem that is also prevalent in other biomedically and ecologically important microorganisms [4,5].

Directly observing complete life cycles can offer a solution to problems such as confusing life cycle stages from the same organism as different species [6,7] and the incorrect identification of sexual stages [8], and could help reveal cryptic microbial life cycle transitions [4,7].

A solution to directly observe life cycle transitions is to use microfluidic devices coupled to time-lapse microscopy, which has been used to directly visualize entire asexual bacterial [9,10] and eukaryotic [11] life cycles in living single cells. However, most experiments deal with short-term processes, especially since the long-term imaging of cells requires experimental conditions for achieving complex cellular transitions that can take days.

To enable long-term imaging of life cycle transitions, we designed this protocol to induce the entire sexual life cycle of *S. cerevisiae*, from proliferation into meiosis, germination, mating, and the proliferation of descendant cells across generations of sexually reproducing diploids. We used a commercially available microfluidic device and a combination of media and micro-culturing conditions that promote transitions across each stage of the yeast life cycle, with minimal perturbation to single cells, and for up to three sexually reproducing generations [12].

This protocol has the potential to enable a complete characterization of the life cycle of yeast cells and to quantitatively study other microbial life cycles, which is crucial to understanding ecological and evolutionary processes in microbial parasites and pathogens [13,14].

## Materials and reagents

**Biological materials**

1. *Saccharomyces cerevisiae* Meyen ex E.C. Hansen 204722^TM^ ATCC SK1 MATa/MATalpha HO can1(r) gal2 cup(s). Wild type prototroph (HIS3/HIS3, TRP1/TRP1, LEU2/LEU2, URA3/URA3) [15]

**Reagents**

1. Adenine hydrochloride hemihydrate (Tokyo Chemical Industry Co., LTD., CAS number: 2922-28-3)

2. Uracil (Tokyo Chemical Industry Co., LTD., CAS number: 66-22-8)

3. L-Histidine (Spectrum Chemical MFG Corp, catalog number: H1021, CAS number: 71-00-1)

4. L-Leucine (Research Products International, catalog number: L22000-100.0, CAS number: 61-90-5)

5. L-Tryptophan (Tokyo Chemical Industry Co., LTD., CAS number: 73-22-3)

6. Arginine hydrochloride (Spectrum Chemical MFG Corp, catalog number: A1337, CAS number: 1119-34-2)

7. L-Aspartic acid (Sigma-Aldrich, catalog number: A9256-100G, CAS number: 56-84-8)

8. L-Glutamic acid (Tokyo Chemical Industry Co., LTD., CAS number: 56-86-0)

9. L-Phenylalanine (Tokyo Chemical Industry Co., LTD., CAS number: 63-91-2)

10. L-Lysine hydrochloride (Research Products International, catalog number: L37040-500.0, CAS number: 657-27-2)

11. L-Methionine (Sigma-Aldrich, catalog number: M5308-25G, CAS number: 63-68-3)

12. L-Serine (Thermo Scientific Chemicals, catalog number: 132661000)

13. L-Threonine (Research Products International, CAS number: 72-19-5)

14. L-Valine (Tokyo Chemical Industry Co., LTD., CAS number:72-18-4)

15. L-(-)-Tyrosine (Tokyo Chemical Industry Co., LTD., CAS number: 60-18-14)

16. L-Isoleucine (Tokyo Chemical Industry Co., LTD., CAS number:73-32-5)

17. Lithium chloride (LiCl) (Sigma-Aldrich, catalog number: L9650-500G, CAS number: 7447-41-8)

18. D(+)-Glucose, ACS reagent, anhydrous (Thermo Scientific Chemicals, catalog number: 410950050, CAS number: 50-99-7)

19. Succinic acid (Research Products International, catalog number: S42000, CAS number: 110-15-6)

20. Sodium hydroxide (Spectrum Chemical MFG Corp, CAS number: 1310-73-2)

21. Ammonium sulfate (Research Products International, catalog number: A20510, CAS number: 7783-20-2)

22. Yeast nitrogen base without amino acids and ammonium sulfate (Sigma-Aldrich, catalog number: Y1251)

23. Bacto^TM^ yeast extract (Gibco, catalog number: DF0127-17-9)

24. Bacto^TM^ peptone (Gibco, catalog number: 211677)

25. Agar, powdered bacteriological grade (Apex BioResearch Products, catalog number: 20-273)

26. Potassium acetate (Research Products International, catalog number: P42025-500.0, CAS number: 127-08-2)

27. Nystatin, powder (Sigma, CAS number: 1400-61-9)

28. Sodium carbonate (Na_2_CO_3_) (Spectrum Chemical MFG Corp., catalog number: S1226, CAS number: 497-19-8)

29. Sorbitol (Sigma-Aldrich, catalog number: S1876-1KG)

30. Glycerol (Sigma-Aldrich, catalog number: G9012-2L, CAS number: 56-81-5)

31. Dimethyl sulfoxide (DMSO) (Fisher Scientific, catalog number: BP231-100)

**Solutions**

1. 4 M LiCl solution (see Recipes)

2. 20% glucose solution (see Recipes)

3. 2% sorbitol solution (see Recipes)

4. 0.25 M Na_2_CO_3_ solution (see Recipes)

5. Synthetic complete dropout powder (see Recipes)

6. Synthetic complete dextrose liquid medium (SCD) (see Recipes)

7. YPD liquid media (see Recipes)

8. YPD plates (see Recipes)

9. Life cycle sporulation liquid media (LC-SPO) (see Recipes)

10. Stock Nystatin solution in DMSO (see Recipes)

**Recipes**

**1. 4 M LiCl solution**

**Table:** 

Reagent	Final concentration	Quantity or volume
Lithium chloride	4 M	17.0 g
diH_2_O	–	100 mL (fill up to)
Total	n/a	100 mL

Prepare 100 mL of 4 M LiCl stock solution by adding 17.0 g of LiCl powder to a sterile 100 mL bottle containing 50 mL of sterile deionized water (diH_2_O). Allow the LiCl to fully dissolve and bring the final volume to 100 mL with sterile diH_2_O. Store at room temperature.

**2. 20% glucose solution**

**Table:** 

Reagent	Final concentration	Quantity or volume
Glucose	20%	200 g
diH_2_O	–	1,000 mL (fill up to)
Total	n/a	1,000 mL

Prepare 1 L of stock 20% glucose solution by adding 200 g of glucose powder to a 1 L flask or beaker containing 500 mL diH_2_O, stirring with a stir bar on a stir plate. Allow the glucose to fully dissolve, remove the stir bar, and bring the final volume to 1 L with diH_2_O. Filter-sterilize using a 0.2 μm Nalgene^®^ bottle-top sterile connected to a vacuum line. See sterilization protocols in Table 1.

**Table 1.** Sterilization protocols

Solution	Sterilization
20% glucose; 2% sorbitol	Filter-sterilize using a 0.2 μm pore size Nalgene^®^ bottle-top sterile filter connected to a vacuum line.
YPD liquid (without glucose)	Autoclave using the 30-min liquid cycle in PRIMUS and Amsco autoclave systems: 121 °C, 15 psi, 30 min.
YPD agar solid (without glucose)	
LC-SPO (without Na_2_CO_3_)	

**3. 2% Sorbitol solution**

**Table:** 

Reagent	Final concentration	Quantity or volume
Sorbitol	2%	20 g
diH_2_O	–	1,000 mL (fill up to)
Total	n/a	1,000 mL

Prepare 1 L of 2% sorbitol solution by adding 20 g of sorbitol powder to a 1 L flask or beaker containing 500 mL of diH_2_O, stirring with a stir bar on a stir plate. Allow the sorbitol to fully dissolve, remove the stir bar, and bring the final volume to 1 L with diH_2_O. Filter-sterilize using a 0.2 μm Nalgene^®^ bottle-top sterile connected to a vacuum line (see Table 1).

**4. 0.25 M Na_2_CO_3_ solution**

**Table:** 

Reagent	Final concentration	Quantity or volume
Na_2_CO_3_	0.25 M	2.65 g
diH_2_O	–	100 mL (fill up to)
Total	n/a	100 mL

Prepare 100 mL of 0.25 M Na_2_CO_3_ solution by adding 2.65 g of Na_2_CO_3_ powder to a sterile 100 mL bottle containing 50 mL of sterile diH_2_O. Allow the Na_2_CO_3_ to fully dissolve and bring the final volume to 100 mL with sterile diH_2_O. Store at room temperature.

**5. Synthetic complete dropout powder**

**Table:** 

Reagent	Final concentration	Quantity
Adenine hydrochloride hemihydrate	2.91%	1.6 g
Uracil	1.40%	0.8 g
L-Histidine	1.40%	0.8 g
L-Leucine	8.70%	4.8 g
L-Tryptophan	5.80%	3.2 g
Arginine hydrochloride	1.40%	0.8 g
L-Aspartic acid	7.20%	4.0 g
L-Glutamic acid	7.20%	4.0 g
L-Phenylalanine	3.60%	2.0 g
L-Lysine hydrochloride	2.20%	1.2 g
L-Methionine	1.40%	0.8 g
L-Serine	27.10%	15.0 g
L-Threonine	14.40%	8.0 g
L-Valine	10.80%	6.0 g
L-(-)-Tyrosine	2.20%	1.2 g
L-Isoleucine	2.20%	1.2 g
Total	n/a	55.4 g

Weigh, combine, and grind the powders of the amino acids described in the table with a clean mortar and pestle until the powder mixture is homogeneously white. Store at room temperature and away from sunlight in an amber bottle.

**6. Synthetic complete dextrose liquid media (SCD)**

**Table:** 

Reagent	Final concentration	Quantity or volume (for 1 L)
Succinic acid	10 g/L	10 g
Sodium hydroxide	6 g/L	6 g
Ammonium sulfate	5 g/L	5 g
Yeast nitrogen base without amino acids and ammonium sulfate	1.7 g/L	1.7 g
Complete dropout powder	1.13 g/L	1.13 g
diH_2_O	–	900 mL (fill up to)
20% glucose (optional, depending on storage)	2%	100 mL
Total	n/a	1,000 mL

Prepare 1 L of SCD liquid media by adding all components in the order described in the table to 500 mL of diH_2_O. Allow each component to fully dissolve before adding the next one by stirring on a stir plate with a stir bar. Remove the stir bar and bring the volume to 900 mL. Split the liquid evenly between two 1 L flasks and autoclave (see Table 1). Store the autoclaved medium at room temperature or 4 °C. Add 100 mL of the filter-sterilized glucose solution, which brings the glucose concentration to 2% and the volume to 1 L. Store at 4 °C.

**7. YPD liquid media**

**Table:** 

Reagent	Final concentration	Quantity or volume (for 1 L)
Bacto^TM^ yeast extract	10 g/L	10 g
Bacto^TM^ peptone	20 g/L	20 g
diH_2_O	–	900 mL (fill up to)
20% glucose	2%	100 mL
Total	n/a	1,000 mL

Prepare 1 L of YPD liquid media by adding all components in the order described to 500 mL of diH_2_O. Allow each component to fully dissolve before adding the next one by stirring on a stir plate with a stir bar. Remove the stir bar and bring the flask to 900 mL. Split the liquid evenly between two 1 L flasks and autoclave for 30 min using the liquid autoclaving cycle (Table 1). When the medium cools down to 40–50 °C, add 100 mL of the filter-sterilized glucose solution and carefully shake, which brings the glucose concentration to 2% and the volume to 1 L. Store at RT.

**Critical:** The glucose solution or media containing glucose should not be autoclaved to prevent caramelization.

**8. YPD agar plates**

**Table:** 

Reagent	Final concentration	Quantity or volume (for 1 L)
Bacto^TM^ yeast extract	10 g/L	10 g
Bacto^TM^ peptone	20 g/L	20 g
Agar, powdered bacteriological grade	20 g/L	20 g
diH_2_O	–	900 mL (fill up to)
20% glucose	2%	100 mL
Total	n/a	1,000 mL

Prepare 1 L of YPD liquid media by adding all components in the order described in the table to 500 mL of diH2O. Allow each component to fully dissolve before adding the next one by stirring on a stir plate with a stir bar. Remove the stir bar and bring the flask to 900 mL. Split the liquid evenly between two 1 L flasks and autoclave for 30 min using the liquid autoclaving cycle (see Table 1). When the medium cools down to 40–50 °C, add 100 mL of the filter-sterilized glucose solution and carefully shake, which brings the glucose concentration to 2% and the volume to 1 L. Pour the hot media into sterile Petri dishes (25 mL per plate) using sterile technique.

**Caution:** Use insulated gloves to handle the hot flasks when pouring the plates. Allow the plates to cool and dry overnight and store at 4 °C.

**9. Life cycle sporulation liquid media (LC-SPO)**

**Table:** 

Reagent	Final concentration	Quantity or volume (for 1 L)
Potassium acetate	6 g/L	6 g
Sorbitol (from 2% w/v stock solution in diH_2_O)	0.02%	20 mL
Adenine hydrochloride hemihydrate	40 mg/L	40 mg
Uracil	40 mg/L	40 mg
L-Histidine	20 mg/L	20 mg
L-Leucine	20 mg/L	20 mg
L-Tryptophan	20 mg/L	20 mg
diH_2_O	–	1,000 mL (fill up to)
0.25 M Na_2_CO_3_ solution (add until pH reaches 8.5)	–	–
Total	n/a	1,000 mL

Prepare 1 L of LC-SPO liquid media by adding all components in the order described in the table to 500 mL of diH_2_O. Add the sorbitol volume from a Nalgene-filter-sterilized 2% w/v sorbitol stock solution. Allow each component to fully dissolve before adding the next one by stirring on a stir plate with a stir bar. Remove the stir bar and bring the flask to 1,000 mL. Split the liquid evenly between two 1 L flasks and autoclave for 30 min using the liquid autoclaving cycle (see Table 1). Right before the microfluidic experiment starts, take a 20 mL aliquot of the LC-SPO media in a sterile beaker with a small stir bar and adjust its pH to 8.5 by dropwise adding 0.25 M Na_2_CO_3_ solution while constantly measuring the pH and stirring on a stir plate. Make sure the pH meter electrode is well rinsed with diH_2_O from the potassium chloride storage solution before measuring the pH of the medium. Use the medium immediately; do not store. This medium is a modification of the sporulation medium from Kociemba et al. [16].

**10. Stock Nystatin solution in DMSO**

Prepare 3 mg/mL of Nystatin by adding 30 mg of Nystatin powder to 5 mL of DMSO. Fill up to 10 mL. Once all Nystatin has dissolved, dispense 1 mL aliquots into 1.7 microtubes and store at -20 °C.

**Laboratory supplies**

1. Petri dishes (KORD-Valmark, catalog number: 2900)

2. 15 mL Falcon tubes, bulk (Olympus plastics, catalog number: 28-103)

3. 1.7 mL microtube (Eppendorf tubes), sterile (Olympus plastics, catalog number: 22-284)

4. Inoculation loops 10 μL, flexible, PP 20/peel bag (Fisherbrand^TM^ Disposable Inoculating Loops, catalog number: 22-363-600 or similar)

5. Ergonomic pipette tips for research, 10 μL (Olympus Plastics, catalog number: 24-130RS)

6. Microfluidic plate for haploid yeast cells (4 chamber, 3.5–5 μm) (CellASIC^®^ ONIX2, catalog number: Y04C-02-5PK)

7. Nalgene^®^ bottle-top sterile, capacity 500 mL, pore size 0.2 μm, fits 45 mm bottle neck (Sigma Millipore, catalog number: Z358223)

8. 2 mL external threaded polypropylene cryogenic vial, self-standing with round bottom (Corning, catalog number: 430659)

9. Electrode storage solution, potassium chloride pH kit (OHAUS, catalog number: 01-922-060)

10. Carl Zeiss immersion oil (refractive index = 1.518, fluorescence free, ISO 8036-1/2 certified, catalog number: NC2187819)

11. Creativity Street Modeling Clay 220g-Neon Colors (Creativity Street, a Dixon Ticonderoga Brand formerly known as Pacon, catalog number: PAC4091, UPC # 021196040915)

12. Olympus spectrophotometry cuvettes, semi-micro (Genesee, catalog number: 21-136)

13. KIMTECH SCIENCE^®^ KIMWIPES^TM^ Delicate Task Wipers (KIMBERLY-CLARK PROFESSIONAL, catalog number: 89218-057)

## Equipment

1. Incubator myTemp (Benchmark, model: H2265HC)

2. Nanodrop (Thermo Scientific, model: NanoDrop ONE^C^)

3. Incu-shaker Mini (Benchmark, model: H1001-M)

4. Vortex mixer (Benchmark, model: BV1000)

5. Ultrasonic homogenizer (Benchmark, model: PULSE 150)

6. Benchtop phase microscope (Zeiss, model: Primostar 3)

7. Mini-centrifuge (Prism^TM^ Mini Centrifuge, catalog number: Z763098); this item has been discontinued, but other mini centrifuges will work, e.g., OHAUS^TM^ Frontier^TM^ 5306 Mini Centrifuge, catalog number: S43248

8. Zeiss Observer Z1 microscope controlled by ZEN pro software with the following specs: a motorized stage, temperature control for 25 °C, a 40× Zeiss EC Plan-Neofluar 40× 1.3 NA oil Ph 3 M27 immersion objective, the focusing system Definite Focus 3.0, an AxioCam 712 monochrome camera, and a X-CITE XYLIS XT720S lamp (Excelitas Technologies) as light source; however, any microscope with a phase contrast objective and a multiposition focusing system can be used

9. Aquasearcher^TM^ pH meter (OHAUS, model: a-AB23PH)

10. pH electrode (OHAUS, model: ST320PH/ATC)

11. CellASIC^®^ ONIX2 Microfluidic System (EMD Millipore Corporation, Merck, model: CAX2-S0000)

## Software and datasets

1. Open-Source software 1: Yeastvision, a pip-installable open-source GUI-based framework for deep-learning-enabled segmentation, tracking, and time-series analysis of the full *Saccharomyces cerevisiae* life cycle. Version 0.1.67. https://pypi.org/project/yeastvision/

2. Imaging data showing the execution of the protocol have been deposited to Dryad (Dataset S1): https://doi.org/10.5061/dryad.3bk3j9kw0

## Procedure

**A. Culturing yeast cells to an early stationary phase**

**Figure 1.**
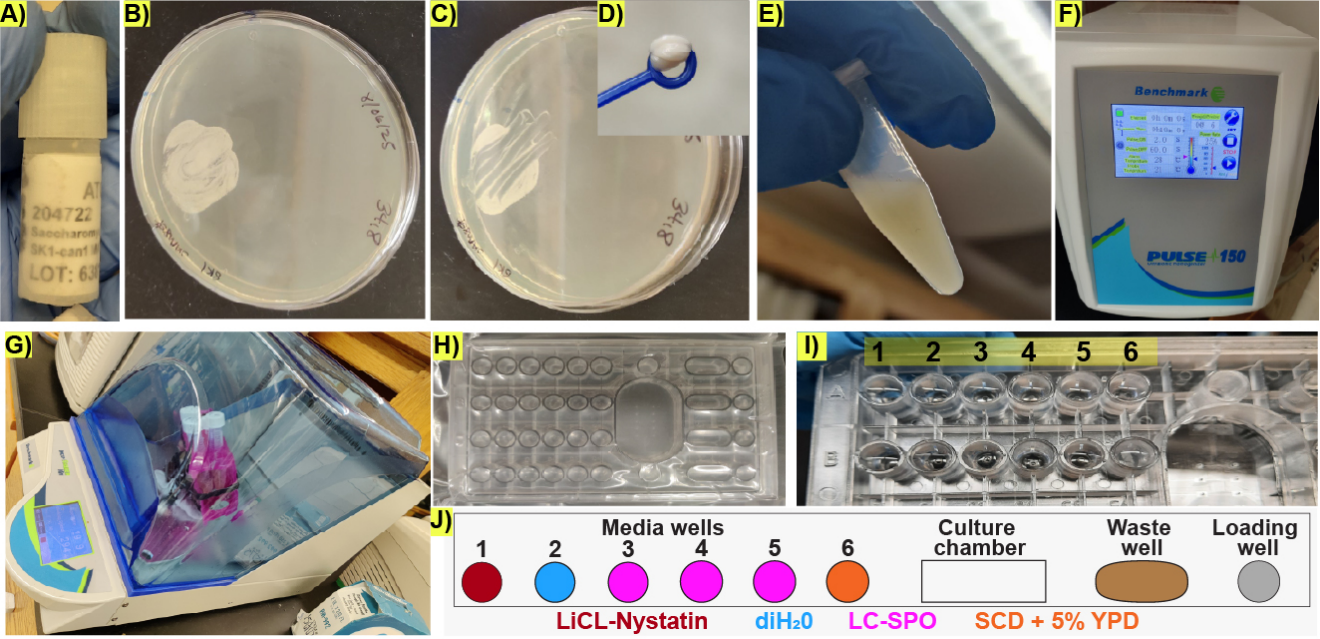
Preparation of early stationary cells for life cycle imaging under microfluidic conditions. (A) Retrieval of frozen stock culture from a -80 °C freezer. (B) Representative image of the patch of cells obtained from the frozen stock culture after overnight incubation at 30 °C. (C) Representative image of the patch of cells after taking a loopful to prepare the dense culture used for liquid inoculation. (D) Representative image of the loopful of cells taken for making a dense cell suspension used for inoculation. (E) Representative image of the dense culture made from the loopful depicted in D. (F) Settings on the ultrasonic homogenizer used for ensuring the cell suspension is homogenous. (G) Arrangement of the 3 mL culture bearing tubes for 23 h incubation. (H) Representative microfluidic plate containing storage liquid buffer in all wells. (I) Representative image of the microfluidic plate media wells after replacing the storage buffer with diH_2_O during rinsing. Numbers indicate the well ID in the CellASIC^®^ ONIX2 user interface. (J) Schematic representation of the arrangement of wells in one lane of the microfluidics device. Colors represent the contents of the well.

1. Retrieve the cryotube containing the diploid yeast strain in 50% glycerol, in this case SK1 (Figure 1A), from the -80 °C freezer.

2. Using a disposable loop, scratch the surface of the frozen yeast stock and patch the diploid yeast strain on a YPD agar plate. Incubate at 30 °C overnight to produce a dense patch of cells (Figure 1B) consisting of budding and unbudded diploid cells, but not sporulating cells, which can be verified by directly observing the cells in a benchtop phase-contrast microscope with 40× magnification.

**Critical:** Avoid leaving the cells on YPD for more than one day because older patches risk unscheduled starvation and accumulating stress-integrating transcription factors such as Xbp1 [17].

3. Take a loopful of cells from the patch (Figure 1C) and resuspend the cells from the overnight patch into 3 mL of SCD medium to an OD_600_ of 0.03–0.06 in a 15 mL Falcon tube following these steps:

a. Prepare a dense stock cell suspension by adding a loopful of cells collected using a 10 μL inoculating loop (Figure 1D) to 750 μL of liquid YPD in a 1.7 mL Eppendorf tube (Figure 1E).

b. Sonicate the cell stock suspension with a 2-s pulse at 25% power using probe 6 on a benchmark pulse 150 ultrasonic homogenizer (Figure 1F). This step can be done with other sonication systems, as long as 95% of the cells remain intact after the sonication step.

c. Add 50 μL of the sonicated cell suspension to 950 μL of liquid YPD in a standard 1 mL spectrophotometry cuvette to obtain a 1:20 dilution of the dense cell suspension.

d. Calculate the amount of volume of the stock cell suspension needed to reach an OD_600_ of 0.06 in the 3 mL culture using the formula (OD_600_ × 20) × V1 = OD_600 _× V2, where (OD_600_ × 20) is the OD_600_ measured for the stock solution times the 1:20 dilution factor, V1 is the volume to be determined, OD_600_ is 0.06, the target OD_600_, and V2 is 3 mL, the target volume. If the calculation results in a V1 smaller than 10 μL of the stock suspension used, dilute the stock suspension by a factor of 10 with YDP and repeat the OD_600_ measurement.

e. Add the calculated volume of stock cell suspension to bring the final volume to 3 mL of YPD with OD_600_ 0.06 in a 15 mL Falcon tube.

f. Place the 15 mL Falcon tube containing 3 mL of cells, at OD_600_ 0.06, in YPD in a 30 °C shaking incubator at 295 rpm at a 45 °C angle (Figure 1G) for 23 h to obtain an early stationary phase culture.

**B. Microfluidic plate preparation**

1. Before the experiment starts: Select a Y04C CellASIC^®^ ONIX2 haploid yeast microfluidic plate with all the wells containing storage buffer, which is included in the microfluidics plate as sold by the vendor (Figure 1H).

**Critical:** If the plate has dry wells, rehydrate the wells for at least 4 h with storage buffer from the other wells or sterile diH_2_O before the experiment begins. Adding media and using dry wells without rehydration introduces air bubbles into the microfluidics device, preventing media flow and negatively affecting pump performance.

2. Using sterile technique and without allowing the wells to dry up, remove the storage buffer with a P-1000 pipette, wash the wells with sterile diH_2_O three times (Figure 1I), and rinse each well with the corresponding medium. Do not allow the wells to dry out between washes. Rinsing is not required for well 1, which is reserved for the LiCl-Nystatin solution.

3. Remove the storage buffer from the culture well and rinse with LC-SPO three times, leaving 100 μL of LC-SPO in the wells to prevent drying while preparing the cells.

4. Load the wells with the corresponding media according to the following order: wells 3–5 contain 290 μL of LC-SPO medium; well 6 contains SCD supplemented with 5% v/v of liquid YPD; well 2 contains sterile diH_2_O; and well 1 contains 100 μL of the 4 M LiCl and 3 μL of the 3 mg/mL Nystatin in DMSO stock solution added right before the experiment starts. This brings the concentration of Nystatin to 0.09 mg/mL in the well.

**Critical:** Because all wells with the same number will be activated simultaneously by the pump (Figure 1I), make sure all other wells contain at least 290 μL of the storage buffer solution or the corresponding media; otherwise, some wells might dry up before the experiment ends. In the same manner, remove the storage buffer from all waste wells, leaving only enough buffer to evenly cover the bottom; otherwise, they might fill up before the experiment ends. The storage buffer can be substituted with sterile diH_2_O to bring unused wells to 290 μL. During the experiment, media will flow through all wells in the plate and will accumulate in all waste wells. A drying media well or an overflowing waste well will produce changes in pressure across the plate and/or a backflow of waste products that could damage the pump/plate and stress the cells.

**C. Cell suspension preparation and plate inoculation**

**Figure 2.**
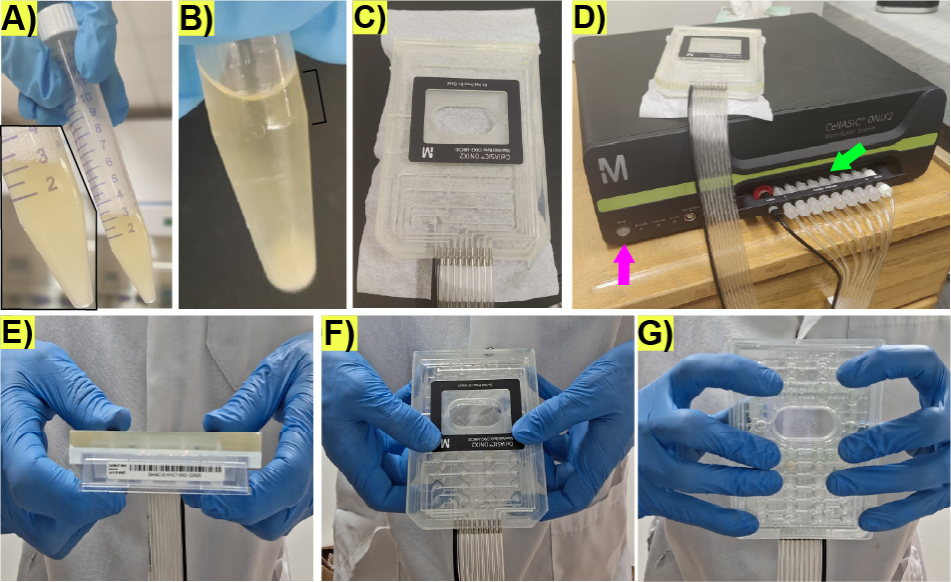
Preparation of the microfluidic plate-manifold setting for microfluidic life cycle imaging. (A) Representative image displaying the culture turbidity, foam, and appearance after overnight shaking incubation and before opening the screw cap. Inlet shows a zoomed in view of the culture in the 15 mL Falcon tube. (B) Representative image displaying the culture after spinning down on a benchtop mini-centrifuge; notice the clumps at the bottom of the tube and the clear appearance of the top from which the cells for loading the microfluidics plate will be taken (black bracket). (C) Representative image of the manifold showing the connections to the vacuum lines. (D) Microfluidic pump controller. The pink arrow represents the “seal button” to trigger the vacuum on the microfluidic plate when attached to the manifold. The green arrow represents the ports for the vacuum lines that should be screwed. (E) Representative image depicting the manifold (top) and the microfluidic plate in contact during sealing. (F) Representative image depicting the top view of finger arrangement to hold the microfluidic plate during sealing. (G) Representative image depicting the bottom view of the finger arrangement to hold the microfluidic plate during sealing.

1. After 23 h of incubation, the 15 mL Falcon tube should contain foam and be under pressure, which is released when opening the screw cap the first time (Figure 2A). Early stationary phase can be confirmed by measuring an OD_600_ of 1.0–1.3.

2. Take 900 μL of cell culture into a 1.7 mL Eppendorf tube. Spin down cell suspension in a Prism^TM^ benchtop mini-centrifuge for 3 s to sediment cell clumps (Figure 2B).

3. Visualize the cells in the upper 200 μL of the spun-down culture in the Eppendorf tube by preparing a slide with 3 μL of culture without disturbing the pellet.

**Critical:** Check that ≥80% of cells in the upper 200 μL of the spun-down culture sample are unbudded and not contaminated. If clumps are still visible, repeat spinning and microscope checking. If ≥20% of cells are budding, culturing conditions may need to be modified to bring cells to early stationary phase, e.g., extending the time the cells are cultured in liquid YPD medium. The upper 200 μL of the spun-down culture should contain small unbudded daughter cells that are easier to load into the microfluidic device compared to large mother cells.

4. Remove sporulation medium from the loading well and add 70 μL of the top 200 μL of the spun-down culture (Figure 2B).

**D. Plate sealing**

1. Place the manifold (Figure 2C) on the microfluidic plate. Ensure there are no tiny objects, such as hair, fibers, lint, or dust, at the interface between the microfluidic plate and the manifold, which can prevent the generation of the vacuum necessary to drive the flow in the wells.

2. Ensure the vacuum lines going into the manifold are properly screwed to the vacuum ports (Figure 2D, green arrow).

3. Trigger the vacuum by pressing the *Seal* button on the pump controller (Figure 2D, pink arrow); immediately press the manifold against the microfluidic plate (Figure 2E) by using your hands as clamps with the thumbs placed on the top of the manifold (Figure 2F) and the fingers evenly distributed on the bottom of the microfluidic plate without touching the microfluidic chambers area (Figure 2G). Maintain physical grip of the plate for 2–3 s after the plate is sealed. The noise coming from the pump during the sealing process should stop in less than 10 s if successful. The noise coming from the activation of the pump controller should be audible at intervals longer than 4 min afterward.

**Critical:** If the pump controller makes noise at intervals faster than 4 min, there might be pressure leakage in the manifold–microfluidic plate interface, and sealing should be repeated. If sealing fails altogether, ensure the manifold–microfluidic plate interface has no dust, debris, or other interfering small objects. If necessary, wipe the manifold–microfluidic plate interface with 70% ethanol and a Kimwipe^TM^ to remove dust and repeat the sealing process. When cleaning with ethanol, make sure pipes in the manifold and the manifold surface are dry, and ethanol does not go into the vacuum lines. Use an empty wash bottle to suction and remove any trapped ethanol. Without a properly sealed manifold–microfluidic plate interface, the experiment might work for the first hours, but the pressure might eventually drop, backflow might occur, or flow might stop.

**E. Mounting the microfluidic plate on the motorized stage**

**Figure 3.**
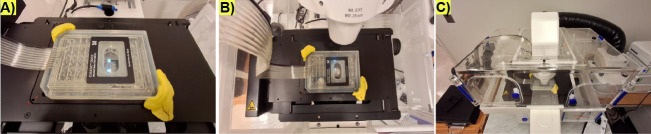
Microfluidic plate-manifold assembly on the microscope stage for long-term life cycle imaging. (A) Representative image depicting how the plate-manifold is held on the stage by yellow modeling clay on the corners. (B) Top view of the arrangement of the plate, including the vacuum lines on the microscope stage and the objective lens under the microfluidic plate. (C) Front view of the full microscope arrangement, including how the vacuum lines are located with respect to the plate to prevent mechanical stress that makes the plate drift over long-term imaging.

1. Open the CellASIC^®^ ONIX2 manifold interface on the control desktop and check that the plate is sealed by looking in the upper left-hand corner. If the plate is sealed, the option to unseal the plate will be displayed.

2. Add Carl Zeiss immersion oil (refractive index = 1.518, fluorescence-free) to the 40× objective. Objectives with higher or lower magnification can be used.

3. (**Critical)** Put the plate in the motorized stage and clamp the corners by using modeling clay to prevent the microfluidic plate from drifting during long-time experiments (Figure 3A). Neodymium magnet-held clamps could replace modeling clay, but modeling clay works fine.

4. Slowly bring the objective lens in contact with the surface of the microfluidic plate (Figure 3B).

**Critical:** The objective should not exert too much pressure (bump) on the microfluidic plate to prevent compromising the integrity of the microchambers. Align the microscope.

5. Make sure the microfluidic vacuum pipelines are in a position that allows the microfluidic plate to move to different fields of view (FOVs) without inducing mechanical stress or strain on the plate and the stage. Notice how in Figure 3C the vacuum lines exit the microscope chamber, making an S-shape with respect to the stage.

**F. Microfluidic protocol programming**

**Figure 4.**
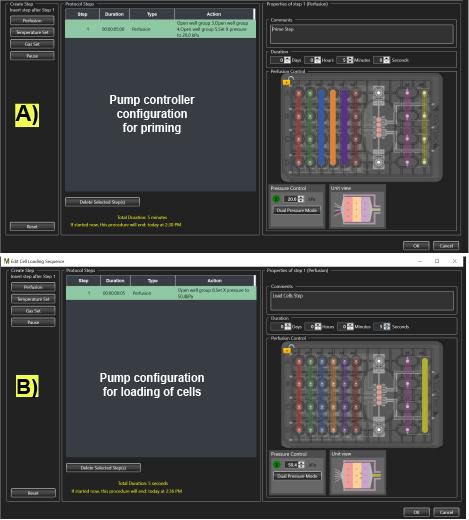
Microfluidic pump controller settings on the CellASIC^®^ ONIX2 program for priming and loading cells in the microfluidic plate. (A) Description of the microfluidic protocol and arrangement of the pump for priming the plate with LC-SPO medium. (B) Description of the microfluidic protocol and arrangement of the pump for loading cells in the microchamber plate. Actively flown wells display solid colors.

**Figure 5.**
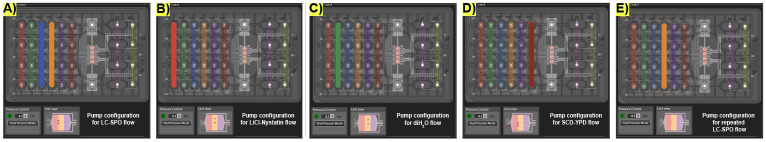
Life cycle induction microfluidic pump controller settings on the CellASIC^®^ ONIX2 program for media switching and rinsing. (A) Arrangement of the pump for the delivery of the LC-SPO medium. (B) Arrangement of the pump for switching to the delivery of Li-Nystatin. (C) Arrangement of the pump for switching to rinsing with diH_2_O. (D) Arrangement of the pump for switching to the delivery of SCD supplemented with 5% YPD. (E) Arrangement of the pump for switching to the delivery of the second round of LC-SPO after the first successful life cycle is completed. Active wells display solid colors.

1. **(Critical)** The following protocols should be pre-programmed in the CellASIC^®^ ONIX2 user interface before the experiment begins:

a. Priming protocol: Activate all wells at 20 kPa for 5 min with all media except LiCl-Nystatin (Figure 4A).

b. Cell loading protocol: Activate the loading cells well at 50.4 kPa for 5 s (Figure 4B).

c. Sporulation microfluidic protocol: Activate LC-SPO-containing wells 3–4 at 8.3 kPa for 48 h (Figure 5A).

d. Spore selection-germination: Activate the LiCl-Nystatin-containing well 1 at 8.3 kPa for 1 h to kill non-sporulated cells (Figure 5B), then activate the diH_2_O-containing well 2 at 8.3 kPa for 1 h to remove the LiCl-Nystatin from the microchamber (Figure 5C), then activate the SCD supplemented with 5% YPD-containing well 6 at 4.2 kPa for at least 8 h to trigger germination and mating (Figure 5D).

e. Repeated sporulation-induction: LC-SPO flows at 8.3 kPa for 48 h from well 5 (Figure 5E).

**G. Priming of the microfluidic plate and focusing with the objective lens**

1. **(Critical)** The storage buffer contained in the microfluidic plate must be removed thoroughly from the microchambers and micropipes. To remove leftover storage buffer from the microchambers and micropipes, activate wells 2–6 for 5 min at 20.0 kPa (step F1a). DO NOT activate the LiCl-Nystatin-containing well 1 because this will prematurely bring LiCl-Nystatin to the microchambers.

2. **(Critical)** Bring the objective to the microfluidics plate surface slowly while focusing on the microchambers’ features. Bumping the objective onto the microfluidic surface might compromise the physical integrity of the microchambers.

**H. Cell loading and imaging**

**Figure 6.**
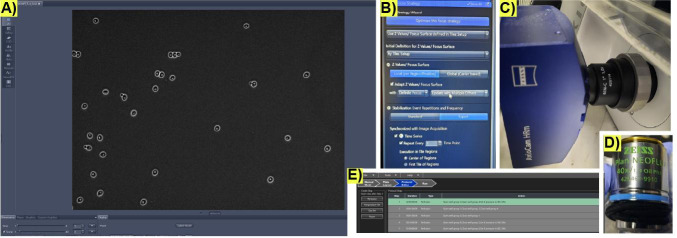
Initial microscopy imaging conditions to record microfluidic cultures for life cycle imaging. (A) Representative image of successfully loaded cells in a microchamber in the microfluidic plate. (B) Settings on the Zen software user interface to program the automated focusing system Definite Focus 3. (C) Monochrome camera and (D) phase-contrast objective used for image acquisition in [12]. (E) Example of all the steps for a full life cycle protocol on the CellASIC^®^ ONIX2 microfluidic interface.

1. Load cells into the microchamber by using the loading option in the CellASIC^®^ ONIX2 interface with a pressure of 50.4 kPa for 5 s (step F1b).

2. Visually check that the entrance to the microchamber is not blocked by cells. The cells that have entered the microchamber must look >85% unbudded and spread out as in Figure 6A. The cells need to be spread out to have enough space to germinate as single microcolonies.

3. If the cell number is too low, repeat loading. If the cells are stuck at the entrance of the microchambers, the microchamber might be damaged, or the cells might be too big to enter it. If too many cells block the entrance to the microchambers, a troubleshooting solution is to repeat the high-pressure priming step F1b, which should dislodge the cells from the entrance.

4. Begin flowing sporulation medium into the cell culture chamber at 8.3 kPa (step F1c) to prevent the cells from staying idle.

5. As fast as possible, select and focus the FOVs to be captured using Zeiss definite focus (Figure 6B), and begin imaging using the Zen software. To replicate the precise images of Kennedy et al. [12], use the phase-contrast objective and camera depicted in Figure 5C, D.

**Critical:** Remember that the objective might apply pressure to the microfluidic plate surface during focusing, causing mechanical stress to the cells.

**Figure 7.**
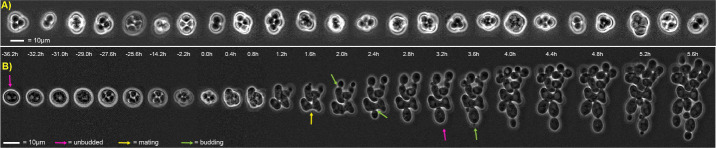
Representative lifecycle imaging time series. (A) Computationally arranged mosaic of examples of mature spores that will resist exposure to the LiCl-Nystatin treatment described in step F1d. (B) Representative sexual life cycle imaging in the model eukaryote *Saccharomyces cerevisiae* using this protocol. Concatenated time series micrographs with computationally aligned yeast cells undergoing a sexual life cycle with cell cycle arrest, sporulation, germination, intra-ascus mating, and proliferation of haploid and diploid (resulting from the mating event) cells. Bar size = 10 μm. Arrows: yellow, mating; pink, unbudded; green, budded cell.

6. Confirm that all FOVs are focused and that the pressure readings are 8.3 kPa ± 0.2 kPa in the CellASIC^®^ ONIX2 interface; deviations from this range might indicate a pressure leak or pressure buildup in the microfluidic plate.

7. Trigger the microfluidic sporulation protocol described in step F1c. The 290 μL LC-SPO-containing wells can support 48 h of imaging at 8.3 kPa, but cells should sporulate in the first 24 h. The period in LC-SPO medium can be extended to 48 h if necessary; however, a lack of sporulation at 24 h indicates that the experiment is unlikely to succeed. Cells should slowly increase in size without cell division during the first 12 h of the experiments if sporulation will occur.

6. Monitor the cells after 24 h and decide whether enough matured sporulated cells [i.e., the ascus has contracted into a tight cruciform, linear, or tetrahedron shape with almost no space between clearly visible spores (Figure 7A)] are present to continue the experiment.

7. End the sporulation microfluidic protocol described in step F1c and use the CellASIC^®^ ONIX2 user interface to start the protocol for killing off non-sporulated cells and triggering the germination and mating of sporulated cells described in F1d.

8. Monitor cell mating and proliferation after exposure to SCD supplemented with 5% YPD; at least 80% of germination events should contain a mating event in the SK1 WT strain. Once the mating cells produce diploid progeny, the experiment will have accomplished the goal of imaging a full lifecycle of yeast from one diploid generation to the next one (Figure 7B). Figure 5 and Table 2 provide examples of protocols that would induce a full life cycle. The sporulation step is set to 48 h just to provide enough time to discern the mature sporulated cells before activating the killing of non-sporulated cells and germination steps (Video 1). Notice that dead cells will remain in the microfluidic chamber, but do not affect living cells.

**Table 2.** Microfluidic life cycle induction steps for early stationary phase *S. cerevisiae* diploids. Diagram of the loaded media described in Figure 1I, J.

Step	Medium	Initial medium volume per well (μL)	Pressure		Temperature (°C)	Hours	Well number on the microfluidic plate
			**psi**	**kPa**			
Sporulation	LC-SPO	290	0.6	8.3	25.0	24–48	2–4
Spore selection	LiCl/Nystatin	103	0.6	8.3	25.0	1.0	1
LiCl/Nystatin rinsing	diH_2_O	290	0.6	8.3	25.0	1.0	2
Germination-mating	SCD + 5% YPD	290	0.3	4.2	25.0	8–12	6

**Video 1.**
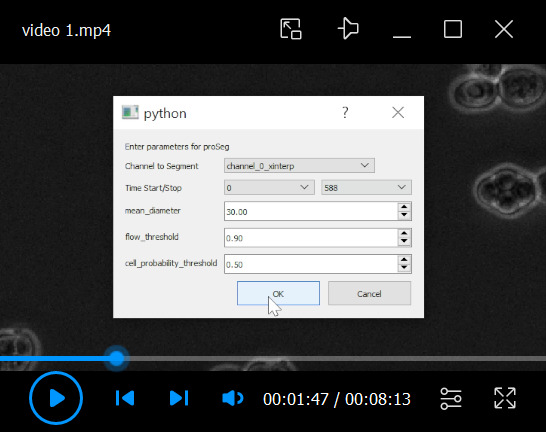
Entire life cycle of the microbe *S. cerevisiae* (yeast)

9. To trigger another consecutive life cycle, the previous microfluidic protocol is stopped after the new diploid generation undergoes only one or two divisions to prevent cells from saturating the microfluidic chamber; the microfluidic sporulation protocol described in step F1e is triggered, followed by the protocol for killing quiescent cells and germination described in step F1d.

**I. Imaging acquisition setup and life cycle capture**

1. Collect images using your microscopy software of choice; we use Zen from Zeiss. The critical component is the *Definite Focus* module that allows the user to take a time series of multiple FOVs. Follow the instructions of your manufacturer to ensure the time series will be focused.

## Data analysis

Several segmentation and tracking algorithms are available to detect and track single cells, e.g., btrack [18]. However, we recommend our Python package Yeastvision, which we developed specifically to track yeast cells during their life cycles and contains pretrained models for life cycle–specific detections on phase-contrast micrographs. The package also offers generative frame interpolation by implementing RIFE (real-time intermediate flow estimation) [19] to ease tracking. All segmentation models can be retrained using this interface to track other types of cells.

## Validation of protocol

This protocol was repeatedly used in the following peer-reviewed research article:

• Kennedy et al. [12]. Deep learning-driven imaging of cell division and cell growth across an entire eukaryotic life cycle. Mol Biol Cell, mbcE25010009. https://doi.org/10.1091/mbc.E25-01-0009

The study shows that this protocol can be combined with fluorescence imaging.

## General notes and troubleshooting

**General notes**

1. Turn on the microscope and the microfluidic pump at least 1 h before starting the experiment to assess whether the pump controller and microscope work correctly.

2. Monitor imaging during the first hour of acquisitions to ensure the FOVs remain focused, the microfluidics plate does not drift, and that the pump maintains constant pressure.

3. Although the Y04C microfluidic plate is marketed for haploid cells, it will work for unbudded diploid cells at the beginning of their cell cycle, mating haploid cells, and proliferating haploid cells. The Y04D plates for diploid cells will not hold all cells in place.

4. While the pump can be programmed with the complete sequence of media switches for triggering all transitions (Figure 6E), we recommend frequently checking the status of the cells, especially sporulation and growth after germination, since factors such as the density of the cells could change when it is the optimal point to change the cells to a different medium. For example, when starting the experiment, we recommend to program the pump to run for 48+ hours in LC-SPO medium, since programming for precisely 24 h with an irreversible switch to the LiCl-Nystatin solution will kill all cells if sporulation was delayed; therefore, it is better to program for 48 h, check the cells at 23 h, and decide whether it is pertinent to trigger the LiCl-Nystatin step at 24 h or wait more time to maximize the number of mature spores.

5. Monitor focus during media exchanges.

6. To study processes that take longer than 48 h to complete, such as sporulation at other temperatures or with other strains, the medium flow could be decreased to extend the duration of the media supply.

**Troubleshooting**

**Problem 1:** The plate fails to seal to the manifold.

Possible cause: Dust, debris, or other interfering small objects, such as hair, dust, or lint, may be present on the manifold surface.

Solution: Wipe the manifold–microfluidic plate interface with 70% ethanol to remove dust and repeat the sealing process. **Critical:** When cleaning with ethanol, prior to re-sealing the plate, ensure pipes in the manifold and the manifold surface are dry, and ethanol does not go into the tubing connected to the pump. Use an empty wash bottle to aspirate remaining ethanol that should be completely removed from the microfluidic manifold pipes and tubing before attempting to re-seal the manifold to the plate.

**Problem 2:** The plate fails to seal to the manifold.

Possible cause: The manifold is not properly aligned with the plate.

Solution: Remove and re-position the manifold before re-attempting to seal.

**Problem 3:** The plate fails to seal to the manifold.

Possible cause: The manifold is not functioning.

Solution: Run the manifold and system self-check following the prompts in the CellASIC^®^ ONIX2 interface.

**Problem 4:** Few cells enter the microfluidic chambers.

**Possible cause:** Cells may be blocking entry into the microchamber.

**Solution:** Move the objective around to visualize the chamber. Repeat the high-pressure priming step F1a to dislodge cells from the entrance and repeat cell loading.

**Problem 5:** Cells are arrested (not dividing) but not sporulating after 24 h.

Possible causes: Initially loaded cells were not in the early stationary phase before loading the microfluidic device, or the experiment setup was too stressful, i.e., the objective bumped onto the microfluidic plate while focusing.

Solution: The period in LC-SPO medium can be extended to 48 h if necessary; however, a lack of sporulation at 24 h indicates the experiment is unlikely to succeed. Re-attempt the experiment with a new microfluidic plate.

**Problem 6:** Quiescent cells are not killed during the selection step.

Possible cause: Selection stress was not enough to kill the cells.

Solution: Perform another round of selection and diH_2_O rinsing as described in step F1d.

**Problem 7:** Microfluidic plate drifts.

Possible causes: Microscope stage is not calibrated properly; arrangement of the microfluidic plate and vacuum lines inside the incubation chamber interferes with plate/stage movement; plate is not secure on the stage.

Solution: Recalibrate the stage; adjust the vacuum lines and microfluidic plate position inside the incubation chamber to minimize interference with plate and stage movement; adjust the modeling clay to keep the microfluidic plate stable on the stage. Observe movements of the stage and plate throughout a few acquisition timepoints to best determine what is the most likely cause of the drift.

**Critical:** Unaddressed drift may cause delays in the Definite Focus performance and damage the objective lens.

**Problem 8:** FOV continually goes out of focus.

Possible cause: Air bubbles present in the immersion oil; the microfluidic plate has surface imperfections.

Solution: If bubbles are present in the immersion oil and interfere with focusing, remove the microfluidic plate from the stage, clean the oil from the objective, add new immersion oil, and focus again. If bubbles are not present but the FOV remains out of focus even after repeated efforts to focus, select another FOV.

## Supplementary information

The following supporting information can be downloaded:

1. Dataset S1. Time-lapse microscopy images of *Saccharomyces cerevisiae*'s full life cycle: https://doi.org/10.5061/dryad.3bk3j9kw0.
